# Simvastatin modulates cellular components in influenza A virus-infected cells

**DOI:** 10.3892/ijmm.2014.1761

**Published:** 2014-04-28

**Authors:** PARVANEH MEHRBOD, MOHD HAIR-BEJO, TENGKU AZMI TENGKU IBRAHIM, ABDUL RAHMAN OMAR, MOHAMED EL ZOWALATY, ZAHRA AJDARI, AINI IDERIS

**Affiliations:** 1 Institute of Bioscience, University Putra Malaysia, Serdang, Selangor 43400, Malaysia; 2 Faculty of Veterinary Medicine, University Putra Malaysia, Serdang, Selangor 43400, Malaysia; 3 School of Chemical Sciences and Food Technology, Faculty of Science and Technology, University Kebangsaan Malaysia, Bangi, Selangor 43600, Malaysia

**Keywords:** statin, influenza, cytokine, actin, RhoA, endocytosis, Rab, autophagy, inhibition

## Abstract

Influenza A virus is one of the most important health risks that lead to significant respiratory infections. Continuous antigenic changes and lack of promising vaccines are the reasons for the unsuccessful treatment of influenza. Statins are pleiotropic drugs that have recently served as anti-influenza agents due to their anti-inflammatory activity. In this study, the effect of simvastatin on influenza A-infected cells was investigated. Based on the MTT cytotoxicity test, hemagglutination (HA) assay and qPCR it was found that simvastatin maintained cell viability and decreased the viral load significantly as compared to virus-inoculated cells. The expression of important pro-inflammatory cytokines (tumor necrosis factor-α, interleukin-6 and interferon-γ), which was quantified using ELISA showed that simvastatin decreased the expression of pro-inflammatory cytokines to an average of 2-fold. Furthermore, the modulation of actin filament polymerization was determined using rhodamine staining. Endocytosis and autophagy processes were examined by detecting Rab and RhoA GTPase protein prenylation and LC3 lipidation using western blotting. The results showed that inhibiting GTPase and LC3 membrane localization using simvastatin inhibits influenza replication. Findings of this study provide evidence that modulation of RhoA, Rabs and LC3 may be the underlying mechanisms for the inhibitory effects of simvastatin as an anti-influenza compound.

## Introduction

Influenza A virus (IAV) (family *Orthomyxoviridae*) is the causative agent of significant respiratory infection (1). The global spread of the 2009 pandemic H1N1 (pH1N1) influenza virus emphasized that humans are limited in their effective strategies to control influenza. Vaccines are the best option for prophylaxis of looming influenza pandemics. However, the time that elapses between characterization of the new strain and vaccine production may have devastating consequences (2). Antiviral therapy is therefore imperative in the control of influenza pandemics (3). However, drug-resistant influenza strains to conventional drugs continuously emerge (4–6). Therefore, the development of anti-influenza drugs with broad reactivity against all strains and subtypes is crucial (2,7). As IAVs use host cell machinery to support their replication and transportation inside the host cell (8), new antiviral treatments should focus on overlapping pathways used by host cells and influenza viruses. The advantage of this approach involves decreasing the potential of drug resistance (8,9).

Previous studies have shown that influenza infections lead to uncontrolled elevations of pro-inflammatory cytokines making this infection a strong risk factor for severe complications (10,11). Some of the most important cytokines involved in the above pathway are tumor necrosis factor-α (TNF-α), interleukin-6 (IL-6) (12) and interferon-γ (IFN-γ) (13). These cytokines induce an innate immune system to control the infection. However, hypercytokinemia occasionally occurs which may cause potentially fatal immune reaction (14). Effective alternative therapeutics to vaccines and conventional antiviral drugs can be based on anti-inflammatory and immunomodulatory agents (7).

Statins, which have the ability to inhibit HMG-CoA reductase, are considered to be mediators of direct cell effects beyond their lipid lowering capacity (15–17). Thus, they block downstream molecules such as isoprenoids and lipid groups, which may be key factors for the virus infectivity cycle (18,19). Due to the anti-inflammatory effects statins exert which result in the reduction of inflammatory reactions, they are regarded as being of clinical significance (20–22).

Actin filaments are necessary for transportations of cells. Cytoskeletal changes were reported following interaction with a number of viruses (23–25). These changes were associated with the binding of different proteins that regulate the dynamics of the actin cytoskeleton (26,27). One class of well-known proteins that regulate actin structures is the Rho family GTPase proteins (28–30). RhoA, the best studied member of this family, triggers the progression of different pathogenic processes (31,32). The effect of statin on disassembly of the cytoskeleton affecting post-translational modification of RhoA has also been demonstrated (33,34).

Pathways such as endocytosis and autophagy are also required for efficient transduction within the cells (35,36). Endocytic pathways reach lysosomes (37) as the final destination of autophagosomes for degradation (38). Rab5 and Rab7 are considered reliable markers for early and late endosomes, respectively. Protein conversion as the mechanism of cargo transport between early and late endosomes even of viral particles trafficking has been previously demonstrated (39,40). By contrast, light chain myosin 3 (LC3) is a reliable marker of autophagosome and autolysosome formation (41). During autophagy the level of membrane-associated LC3-II (16 kDa) increases, while the level of soluble LC3-I (18 kDa) decreases (42,43). Therefore, LC3-II levels correlate with the number of autophagosomes (44).

Numerous pathogens including viruses hijack the endocytic and autophagic pathways mediating their internalization as well as trafficking to the site of replication to avoid being degraded by the lysosomes (26,38,39).

To the best of our knowledge, limited data are available on the anti-inflammatory effects and inhibitory role of statins on RhoA, Rab and LC3 protein functions induced by IAV infection. The therapeutic role of statins on influenza infection with regard to their effects on these proteins has not yet been established. In the present study the effects of simvastatin on IAV replication were evaluated using MTT cytotoxicity and hemagglutination (HA) assays. The cytoskeletal effects of simvastatin in the presence of IAV via its effect on RhoA protein prenylation were also investigated. This study characterized the involvement of simvastatin on viral cellular infection via its effect on Rab proteins during endocytosis and its effect on lysosomal activity by affecting autophagosomes. The results suggested that simvastatin should be further investigated in order to be introduced as a potential anti-influenza compound in the future.

## Materials and methods

### Reagents, chemicals and antibodies

Cell culture media and penicillin-streptomycin solution were purchased from Mediatech Cellgro Co. (Northbrook, IL, USA). Accutase cell detachment solution was obtained from Innovative Cell Technologies (San Diego, CA, USA) and fetal bovine serum (FBS) was purchased from PAA Laboratories (Pasching, Austria). Plastic wares were obtained from Orange Scientific (Braine-l’Alleud, Belgium) while 8-well Lab-Tek II Chamber Slides were purchased from Thermo Scientific (New York, NY, USA). Simvastatin base, amantadine hydrochloride, oseltamivir phosphate, RhoA inhibitor Y-27632 dihydrochloride, farnesyl pyrophosphate (FPP), geranylgeranyl pyrophosphate (GGPP), farnesyl transferase inhibitor (FTI-276), geranylgeranyl transferase inhibitor (GGTI-2133), cholesteryl ester, Bafilomycin A1 (BafA1), tosylamide phenylethyl chloromethyl ketone-treated trypsin (TPCK-Trypsin), 3-(4,5-dimethyl-2-thiazolyl)-2,5-diphenyl-2H-tetrazolium bromide (MTT) and BCIP-NBT substrate were obtained from Sigma (St. Louis, MO, USA). Fluorescent rhodamine 110 phalloidin was purchased from Biotium (Hayward, CA, USA) and ProLong^®^ Gold Antifade reagent and LysoTracker Red DND-99 were purchased from Invitrogen (Carlsbad, CA, USA). CytoBuster™ protein extraction reagent was provided from Novagen (Madison, WI, USA). Protein extraction kit (ab65400), rabbit primary polyclonal anti-RhoA (ab68826), donkey secondary polyclonal anti-rabbit (AP-conjugated) (ab97061), rabbit primary polyclonal anti-Rab5 (ab18211), goat secondary polyclonal anti-rabbit IgG (AP-conjugated) (ab97048), mouse primary monoclonal anti-Rab7 (ab50533), goat secondary polyclonal anti-mouse IgG (AP-conjugated) (ab97020), rabbit primary polyclonal anti-LC3 (ab58610), mouse primary monoclonal anti-GAPDH (ab9484), mouse primary monoclonal anti-pan-Cadherin (ab6528) and goat secondary polyclonal anti-mouse (AP-conjugated) (ab97020) were purchased from Abcam (Cambridge, UK). Simvastatin (10 mg) was dissolved in 1 ml of DMSO while Y-27632 (10 mg) was dissolved in 1 ml of dH_2_O. FTI-276 and GGTI-2133, which served as control drugs for simvastatin, were also dissolved in DMSO. Amantadine hydrochloride, which served as the control antiviral drug, was dissolved in dH_2_O. The stock solutions were filter-sterilized using 0.22 μm syringe filter, aliquoted and stored at −20°C. Control experiments were carried out using solvents but no effects on these vehicles were observed.

### Virus and cell culture

Influenza virus strain A/New Jersey/8/76 (H1N1) [A/NJ (H1N1)] (ATCC VR-897™) was used in the present study. The viral stock was propagated in Madin Darby canine kidney (MDCK) cells in the presence of 1 μg/ml of trypsin-TPCK. The MDCK cell line was purchased from ATCC (CCL-34™). The cells were grown in Dulbecco’s modified Eagle’s medium (DMEM) containing 10% heat-inactivated FBS, 100 U/ml penicillin G and 100 μg/ml streptomycin. Cultured cells were incubated at 37°C, in a 5% CO_2_ atmosphere. During antiviral evaluations, FBS was washed with phosphate-buffered saline (PBS) and the medium was supplemented with TPCK-treated trypsin (1 μg/ml). For virus-stock preparation, the MDCK cells were infected with the virus at a multiplicity of infection (MOI) of 0.5. Progeny virus was harvested three days post-infection. Standard HA tests using tissue culture infectious dose 50 (TCID_50_) and the Karber formula (45,46) were conducted to measure virus infectivity.

### Cell viability

MDCK cells sub-cultured in 96-well plates were exposed to different concentrations of simvastatin at various time intervals of 24, 48 and 72 h in triplicate. A colorimetric MTT assay was performed as previously described (47). Color adsorption in the solution was measured using a microplate reader (BioTek EL 800; BioTek Instruments Inc., Winooski, VT, USA) at 570 nm. The 50% cytotoxic concentration (CC_50_) causing visible morphological changes in 50% of the cells with respect to cell control, and effective concentration (EC_50_), which is the concentration required to achieve 50% protection against a virus-induced cytopathic effect and cell viability, were defined by this method.

In one series of experiments, MDCK cells were pretreated with FPP, GGPP and cholesterol for a period of 6 h. The cells were then washed with PBS, followed by treatment with simvastatin and H1N1 (100 TCID_50_) for 1 h. The cells were then washed with PBS and subsequently incubated in the medium supplemented with simvastatin and TPCK for 48 h at 37°C. FTI, GGTI, amantadine and oseltamivir treatments were simultaneously examined.

### Hemagglutination assay

MDCK cells were pretreated with FPP, GGPP and cholesterol as previously mentioned for 6 h. Following washing, the combined treatment of simvastatin and H1N1 was conducted for 1 h. The cells were supplemented with simvastatin and TPCK and incubated for a period of 48 h at 37°C. The effects of FTI, GGTI, amantadine and oseltamivir were simultaneously examined. The virus titer was quantified using HA assay from the cell supernatants, as previously described (48). Briefly, serial double dilutions of the culture media were added to 96-well U-shaped microplates in duplicate. HA units were calculated as the reciprocal of the highest dilution, yielding complete agglutination. Chicken red blood cells were used at a concentration of 0.5%.

### RNA extraction and cDNA synthesis

MDCK cells were washed with PBS following treatments with simvastatin (10 μM) and H1N1 (100 TCID_50_) for 1 h. In the co-inoculation treatment, the cells were exposed to simvastatin and H1N1 simultaneously. In the pre-inoculation treatment the cells were initially treated with simvastatin for 1 h, followed by H1N1 inoculation, whereas in the post-inoculation treatment the cells were initially inoculated with H1N1 for 1 h followed by the addition of simvastatin. Following the treatments, the cells were washed again with PBS and incubated with medium supplemented with simvastatin and TPCK for 48 h at 37°C.

Viral RNA was extracted using the Viral Nucleic Acid Extraction kit II (Geneaid, Taipei, Taiwan), as per the manufacturers’ instructions. The extracted RNAs were reverse-transcribed using a RevertAid H Minus First Strand cDNA kit (Fermentas, Burlington, ON, Canada). The incubation cycle was performed as previously described (49). Virus-inoculated and mock-infected supernatants were considered as positive and negative controls, respectively. The gene actin α1 (ACTA1) was targeted as the housekeeping control.

### Quantitative polymerase chain reaction (qPCR)

qPCR assay was carried out on a 10-fold serially diluted positive control for each target gene in order to construct the standard curves. Copy numbers for the standards were calculated as previously described (50). The reaction was carried out with Maxima SYBR-Green/Fluorescein qPCR master mix (Fermentas) using Thermo Cycler Apparatus (Bio-Rad, Philadelphia, PA, USA) in a total volume of 25 μl. The PCR protocol was generated using the software program as previously described (49). Melting curve analysis was performed to confirm the specificity of the amplified products. Data acquisition and analysis were performed using CFX Manager version 2.0. Primer sequences used for M2 amplification were described in a previous study (49). Amplification for the *NP* gene (CY039994) was carried out with NP-A-For primer (CAG ACC AAA TGA AAA CCC AGC) and NP-A-Rev primer (AAT CTG AAC CCC TCT TGT GG) at a 147 bp length from position 973 to 1120 of the *NP* gene. Amplification for the *ACTA1* gene (NM_001195845.1) was carried out with ACTA1-For primer (TTC CGC TGC CCA GAG GCT CT) and ACTA1-Rev primer (GCT CAG GGG GTG CGA TGA TCT TG) for the internal control at a 240 bp length from position 909 to 1249 of *ACTA1* gene.

### Cytokine protein quantification

Confluent monolayer of MDCK cells incubated for 24 h at 37°C, 5% CO_2_ in 96-well flat-bottom tissue culture plate was exposed to 100 TCID_50_ of H1N1 in the presence or absence of simvastatin. Untreated MDCKs were considered as the negative control. Cell-free supernatants were treated for 24, 48 and 72 h at different concentrations in duplicate and then stored at −80°C for the cytokine analysis. Quantitative sandwich ELISA was performed by Quantikine ELISA kits (R&D Systems, Minneapolis, MN, USA) as per the manufacturer’ instructions.

### Immunofluorescent labeling and confocal laser scanning microscopy

MDCK cells were cultured and allowed to grow up to 80% confluency in 8-well chamber-slides (8×10^4^ cell/well) for 24 h. The cells were then treated with simvastatin at a concentration of 10 μM in the presence or absence of H1N1 at a concentration of 100 TCID_50_/0.1 ml. At the end of the incubation period of 24 h, the cells were fixed in 3% paraformaldehyde in PBS for 10 min at 4°C. The cells were then washed with PBS and permeabilized at room temperature with 0.2% Triton-X-100 and blocked with 3% non-fat dry milk in PBS for 1 h at 4°C. The cells were then labeled with rhodamine 110 phalloidin staining at a concentration of 2 μM (1:50 dilution in blocking buffer) for 20 min at room temperature. DAPI (1 μg/ml) was used as the nuclear counterstain. The slide was then mounted with warm anti-fade reagent and sealed with clear lacquer.

Fluorescent images were obtained by confocal microscopy (LSM 518F; Zeiss, Jena, Germany) using suitable filters (excitation at 502 nm and emission at 524 nm for rhodamine 110 phalloidin and excitation at 358 nm and emission at 461 nm for DAPI) and 100× oil immersion lens, pixel resolution of 0.164 μm, scan speed of 5, and four-line averaging from an argon-krypton laser. The micrographs were processed using appropriate software (LSM5) and the average fluorescence intensities in all the treatments as a ratio to the untreated control were used to quantify the intensity results.

### Sample preparation for RhoA, Rab and LC3 protein immunoblotting

MDCK cells were cultured in 75 cm^2^ flasks. To detect prenylated RhoA protein, the cells were simultaneously treated with simvastatin and Y-27632 (10 μM) in the presence or absence of H1N1 (100 TCID_50_/0.1 ml) for 48 h. To detect prenylated Rab proteins, the cells were treated with simvastatin (10 μM) in the presence or absence of H1N1 (100 TCID_50_/0.1 ml) for 48 h. The effects of simvastatin for these detections were disrupted by 6 h pretreatment of exogenous FPP and GGPP (10 μM). To detect LC3 protein, MDCK cells were pretreated with BafA1 (20 nM) as a lysosome activity inhibitor for 6 h with and/or without simvastatin (10 μM) in the presence or absence of H1N1 (100 TCID_50_/0.1 ml) for 48 h.

For all the treatments, the cells were trypsinized, scraped, collected in ice-cold PBS and centrifuged to form pellets. For RhoA and Rabs detection, the cell pellet was re-suspended and homogenized in 1 ml of homogenizing buffer mixed with protease inhibitor cocktail. The homogenate was centrifuged at 700 × g for 10 min, at 4°C. The pellet was then discarded and the supernatant was subjected to high-speed centrifugation at 15,000 × g for 1 h, at 4°C. The pellet (membrane fraction) was dissolved in 100 μl of 0.5% Triton X-100 in PBS. All the membrane and cytosol fractions were stored at −80°C. For LC3 detection, the cell pellet was re-suspended and homogenized in 1 ml of CytoBuster lysis buffer and protease inhibitor cocktail at room temperature. The homogenate was centrifuged at high speed at 15,000 × g for 1 h, at 4°C. The pellet was discarded and the supernatant was stored at −80°C for subsequent use. The protein content in each sample was quantified using the Bradford assay.

### Detection of RhoA and Rabs translocation and LC3 lipidation in MDCK cells

A quantity of 0.5 μg protein of each sample was fractioned using sodium dodecyl sulfate polyacrylamide gel electrophoresis (SDS-PAGE) in 15% mini-gels and transferred onto nitrocellulose membrane (Bio-Rad) by vertical semi-dry electroblotting. Membranes were blocked for 1 h at room temperature using milk diluent as blocking buffer (Kirkegaard and Perry Laboratories, Gaithersburg, MD, USA). Following three washing steps with Tris-buffered saline (TBS)-Tween, the membranes were incubated overnight with primary antibody at 4°C. Following incubation with primary antibody and washing, the membranes were exposed to alkaline phosphatase-conjugated antibody for 1 h at room temperature. Following several washing steps, the enzymatic reaction was visualized using a BCIP-NBT substrate. Antibodies against Pan-cadherin and GAPDH housekeeping proteins were used for membrane and cytosolic loading controls, respectively. Blots were imaged using Odyssey infrared imaging system (Li-COR Biosciences, Lincoln, NE, USA). Quantification was performed on single channels using the analysis software provided in the Odyssey Infrared Imaging System (Li-COR Biosciences, Lincoln, NE, USA). For each sample, signal intensities were normalized to the appropriate internal control.

### Modulation of the lysosome localization in MDCK cells

To investigate lysosomal activity and localization, MDCK cells were cultured in 8-well chamber-slides (8×10^4^ cell/well) for 24 h and further treatment with simvastatin (10 μM) and H1N1 (100 TCID_50_/0.1 ml) was conducted in wet chambers for 24 h. At the end of the incubation period the lysosomal mass was visualized by staining with LysoTracker Red DND-99 probe. Briefly, the cells were washed with PBS. Pre-warmed LysoTracker Red containing media at 50 nM concentration of probe was added to the coverslip for 25 min under growth conditions appropriate for the cell culture in the dark. Loading solution was replaced with fresh media. The cells were fixed with 3% paraformaldehyde for 10 min at room temperature. The slide was washed with PBS, mounted with warm mounting buffer ProLong^®^ Gold anti-fade reagent, sealed with clear lacquer and stored at 4°C the dark overnight. Images were obtained by confocal microscopy (Zeiss LSM 518F; Zeiss) with proper filters (excitation at 577 nm and emission at 592 nm) using 63× objectives (oil, NA=104), pixel resolution of 0.164 μm, scan speed 5 and four-line averaging. Images were processed with the appropriate software (LSM5) and intensity data [mean ± standard deviation (SD)] were used to quantify the results.

### Statistical analysis

Statistical analysis of the data was performed using SPSS 20.0. Data were presented as mean ± SD. A two-way or one-way analysis of variance (ANOVA) post-hoc LSD test was used to analyze the data for cytotoxicity, HA, qPCR and ELISA assays. The Student’s t-test was used to examine the effect of treatments on actin filament polymerization, lysosomal mass localization, RhoA and Rab protein prenylation and LC3 lipidation. For all the tests, P≤0.05 was considered significant.

## Results

### Cell viability

The viability of MDCK cells following 24, 48 and 72 h treatment with different concentrations of simvastatin as determined by MTT assay are shown in Table I. The results showed that simvastatin had no cytotoxic effect on the cell at concentrations up to 15 μM. The EC_50_ of simvastatin was calculated from MTT data using the two-way ANOVA test at a concentration of 10 μM, which had no significant cytotoxic effect on the cell viability as compared to the control.

### Inhibitory effect of statins on the virus

**Figure 1:**
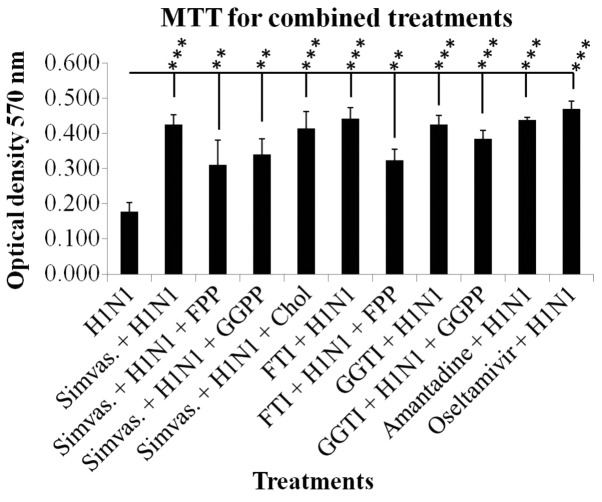
Cell viability test. Optical densities obtained from combined treatments of simvastatin and H1N1 for 48 h are shown as mean ± standard deviation (SD) as the average of three independent experiments in triplicate and expressed as a ratio to the control group. Farnesyl transferase inhibitor (FTI), geranylgeranyl transferase inhibitor (GGTI), amantadine and oseltamivir were also simultaneously tested as control drugs. The simvastatin effect on cell viability was partially disrupted by exogenous farnesyl pyrophosphate (FPP) and geranylgeranyl pyrophosphate (GGPP), although it was not affected by cholesterol. (^***^P≤0.001; ^**^P≤0.01).

Increased optical density in the combined treatments of simvastatin and H1N1 as compared to H1N1 alone was markedly significant (P≤0.001). The effect of simvastatin on cell viability was affected by exogenous FPP and GGPP although this effect was not significant; however, cell viability was not affected by cholesterol (Fig. 1). The significant increase in cell viabilities as compared to H1N1 infection demonstrated the protective effect of simvastatin on the cell viability against viral cytopathic effects. The results of FTI, GGTI, amantadine and oseltamivir treatments are shown in Fig. 1. Treatment with Y-27632 also showed similar results to simvastatin (data not shown).

### Hemagglutination assay results

**Figure 2:**
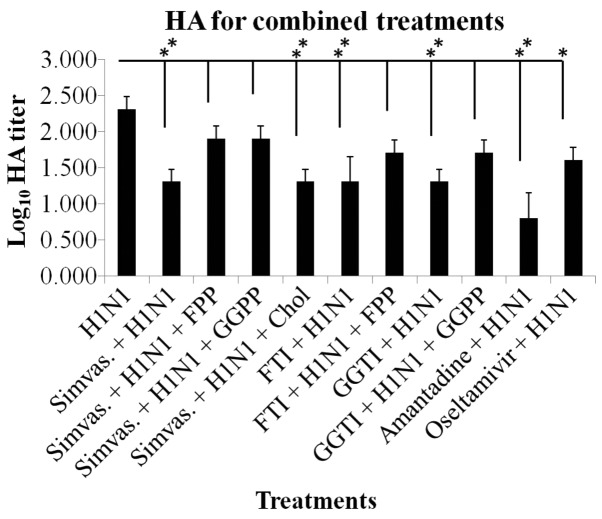
Hemagglutination (HA) assay. Log_10_ HA titers are presented as mean ± standard deviation (SD) as the average of three independent HA titrations in triplicate for combined treatments of simvastatin and H1N1 for 48 h. Farnesyl transferase inhibitor (FTI), geranylgeranyl transferase inhibitor (GGTI), amantadine and oseltamivir were also tested as control drugs. Simvastatin effect on HA titration was partially disrupted by exogenous farnesyl pyrophosphate (FPP) and geranylgeranyl pyrophosphate (GGPP), but not by cholesterol. (^**^P≤0.01; ^*^P≤0.05).

Based on HA titration, the inhibitory effect of simvastatin on viral adsorption to the cell surface was demonstrated by a significant reduction in the HA titer unit in the combined treatments (P≤0.01 and P≤0.05) (Fig. 2). The results of FTI, GGTI, amantadine and oseltamivir treatments are shown in Fig. 2. Y-27632 treatment results were similar to the simvastatin effects (data not shown). Notably, the addition of exogenous FPP and GGPP reversed the effects of simvastatin on HA titration although not significantly, while cholesterol did not interfere with the effects of statins.

### Absolute quantification

Amplification graph and melt curve analysis for each gene amplification confirmed the specificity of the amplified products (data not shown). Quantitative analysis of PCR products of NP and M2 IAV genes at different time points yielded significant decrements in log_10_ copy numbers of viral genes compared to H1N1-inoculated cells (P≤0.01 and P≤0.001). Amantadine treatment was conducted as the control treatment (Table II).

### Quantification of cytokine expression levels by ELISA assay

TNF-α, IL-6 and IFN-γ proteins were not detectable in supernatants of MDCK cell culture at 24 and 48 h after exposure (data not shown). However, at 72 h treatments, H1N1 infection exhibited highly significant expression levels of these cytokines compared to the simvastatin-treated supernatants (P≤0.001). Fold reduction of the protein expression shown in Table III was averaged at 2.5, 1.5 and 2 for TNF-α, IL-6 and IFN-γ, respectively.

### Alterations of actin cytoskeleton organization

**Figure 3:**
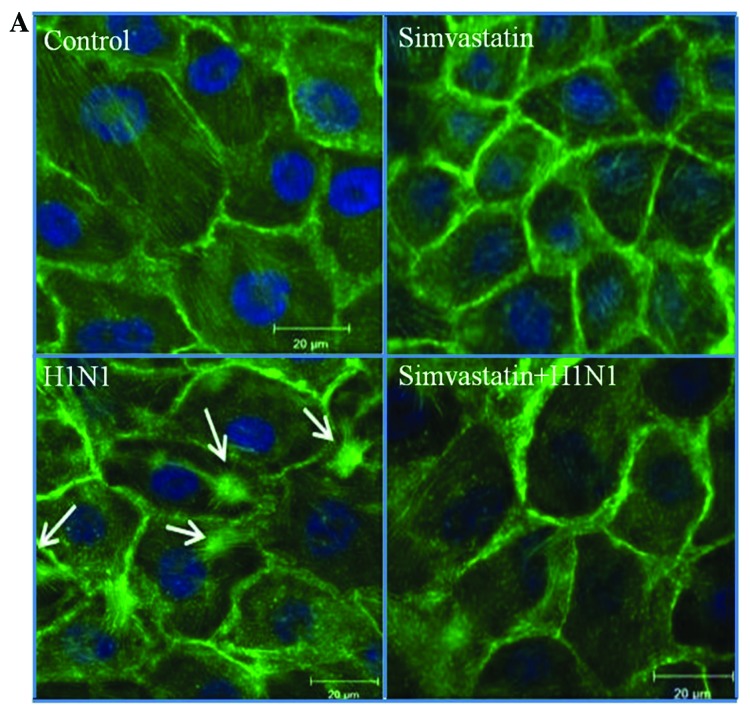

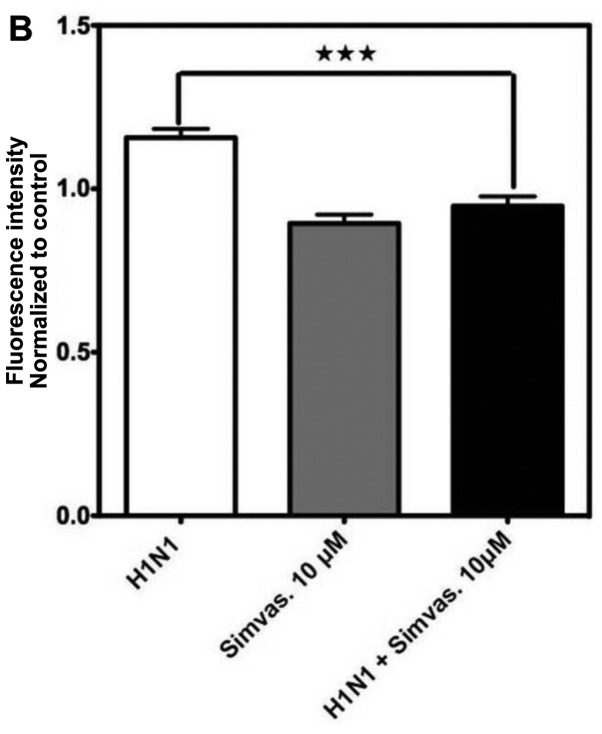
Alterations of actin cytoskeleton organization in Madin Darby canine kidney (MDCK) cell. Images show the effect of simvastatin and H1N1 virus on actin fibers. (A) MDCK cells were stained with phalloidin after culture in the absence of simvastatin and 24 h later following the addition of 10 μM of simvastatin, 100 TCID_50_ influenza A virus and the combined treatments. Control staining shows a normal appearance of actin fibers. Images are representative of 10 independent experiments performed on the cells, with similar results. Arrows show the star-shape of condensed filaments and the panels are of equal magnification. Bar, 20 μm. (B) Fluorescent intensities of actin fibers in MDCK cells following treatment with simvastatin in the presence or absence of influenza A virus treatment for 24 h. Data are averages of 10 independent repeats of intensities [mean ± standard deviation (SD)] normalized to the negative control, evaluated by LSM software and analyzed by one way ANOVA. (^***^P≤0.001).

Images of MDCK cells grown under normal conditions showed spindle-like appearance with numerous distinct and well-organized actin fibers extending across the cytosol to the plasma membrane. Following treatment with simvastatin at a concentration of 10 μM, actin filaments appeared disassembled with loss of correlation between spanning fibers. Infection of MDCK cells with H1N1 showed an increased incidence of actin stress fibers and remodeling of the actin cytoskeleton switching to a condensed star-shape appearance. Images of the combined treatments clearly showed the inhibitory effect of simvastatin on actin filament condensation caused by H1N1 (Fig. 3A). These images were processed with LSM5 software and the average fluorescence intensities of at least 10-fold repetitions (mean ± SD) were used to quantify the results. Data were normalized to the negative control. Statistical analysis of fluorescent intensity for these treatments showed a significant decrement in the combined treatment as compared to H1N1. This difference was highly significant (P≤0.001) (Fig. 3B).

### Simvastatin and H1N1 effects on RhoA and Rabs translocation and LC3 lipidation

**Figure 4:**
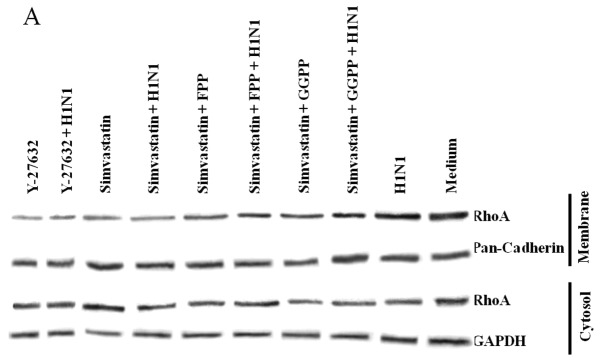

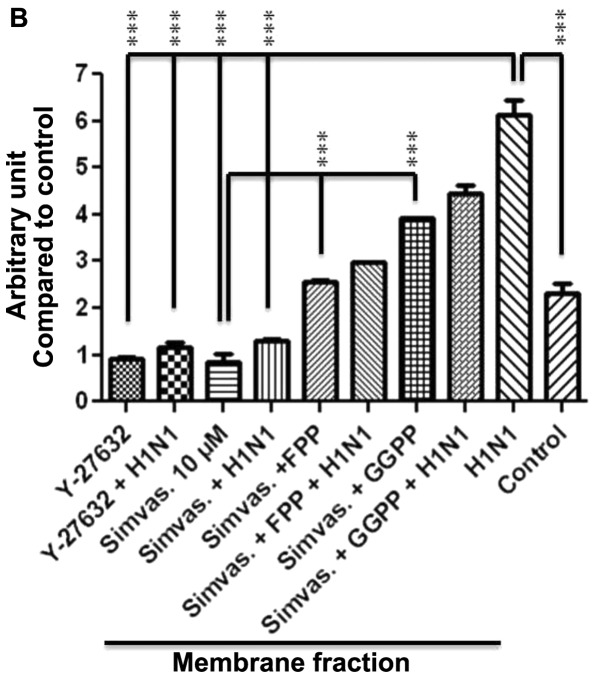

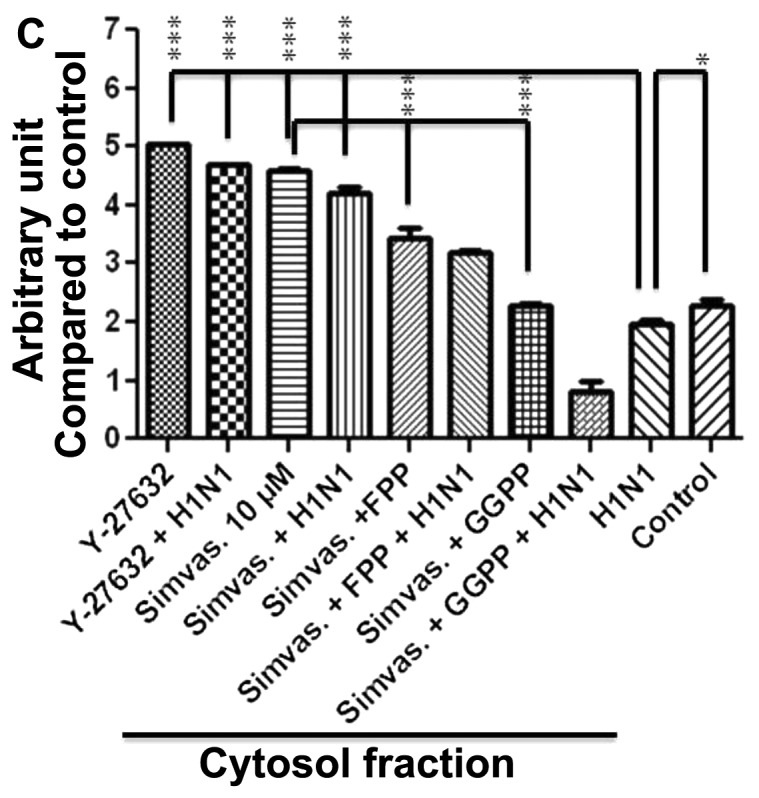
Modulation of the RhoA protein translocation. (A) Confluent Madin Darby canine kidney (MDCK) cells were treated with different simvastatin and Y-27632 in the presence or absence of influenza A virus for 48 h. The effects of simvastatin were disrupted by 6 h pretreatment of exogenous farnesyl pyrophosphate (FPP) and geranylgeranyl pyrophosphate (GGPP). The blots were typical of three independent experiments with similar results. (B and C) Density values of RhoA protein isoprenylation were shown in membrane and cytosol extracts of treatments. Values show the density of the RhoA protein bands normalized to the related housekeeping protein determined by Odyssey Infrared Imaging System (Li-COR Biosciences) and present the mean ± standard deviation (SD) of three different experiments from membrane and cytosol fractions of cell lysates. Data were analyzed statistically by the Student’s t-test. (^***^P≤0.001; ^*^P≤0.05; NS, not significant).

**Figure 5:**
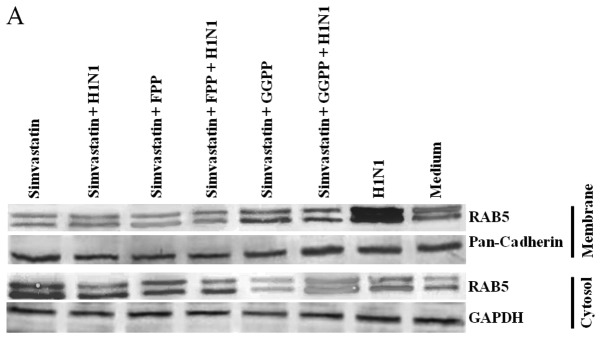

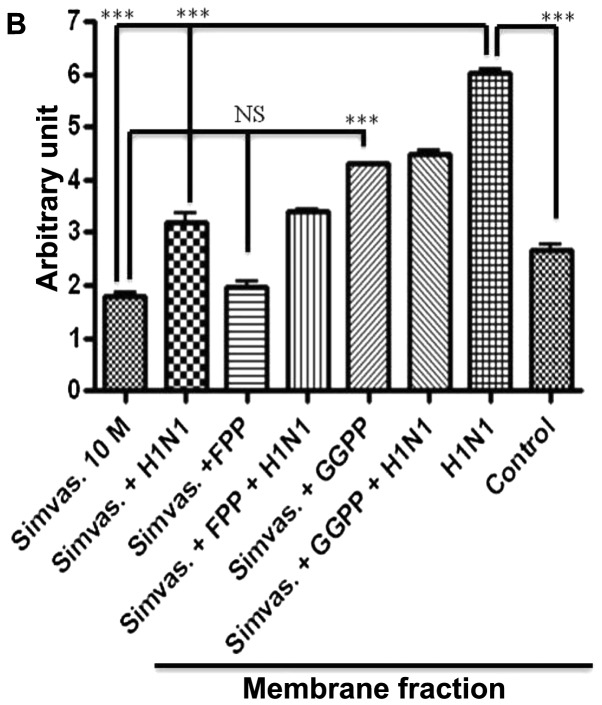

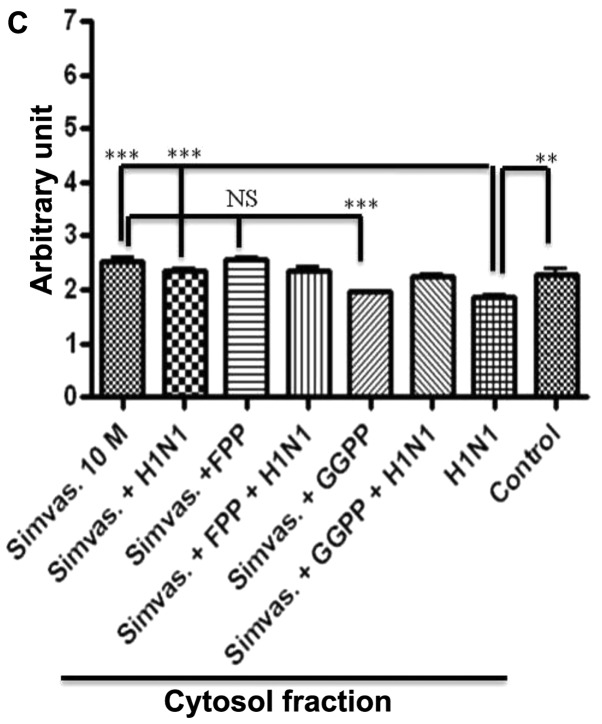
Modulation of the Rab5 protein translocation. (A) Confluent Madin Darby canine kidney (MDCK) cells were treated with simvastatin in the presence or absence of influenza A virus for 48 h. The effects of statin were disrupted by 6 h pretreatment of exogenous geranylgeranyl pyrophosphate (GGPP), but not farnesyl pyrophosphate (FPP). The blots were typical of three independent experiments with similar results. (B and C) Density values of Rab5 protein isoprenylation are shown in the membrane and cytosol extracts of treatments. Values show the density of the Rab5 protein bands normalized to the related housekeeping protein determined by Odyssey Infrared Imaging System and present the mean ± standard deviation (SD) of three different experiments from membrane and cytosol fractions of the cell lysates. Data were analyzed statistically by the Student’s t-test. (^***^P≤0.001; ^**^P≤0.01; NS, not significant).

**Figure 6:**
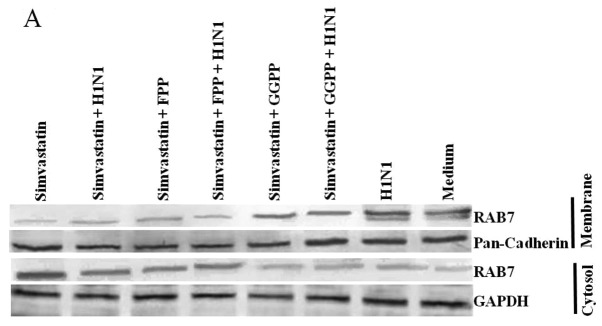

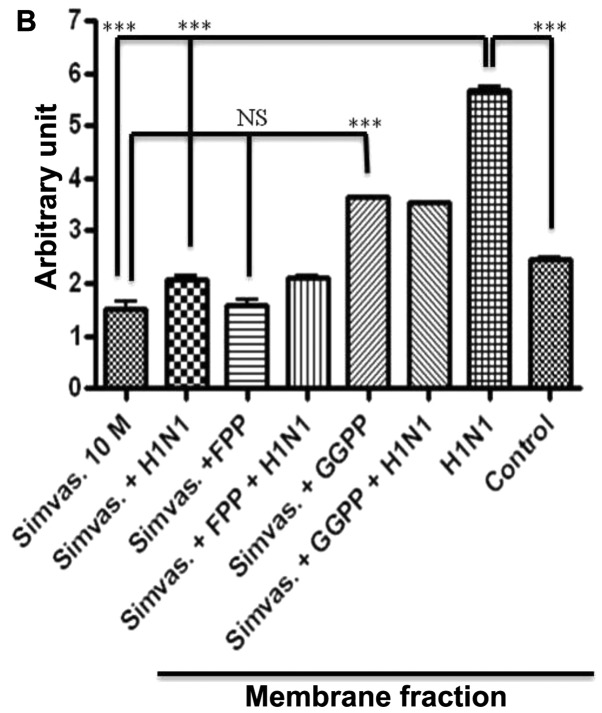

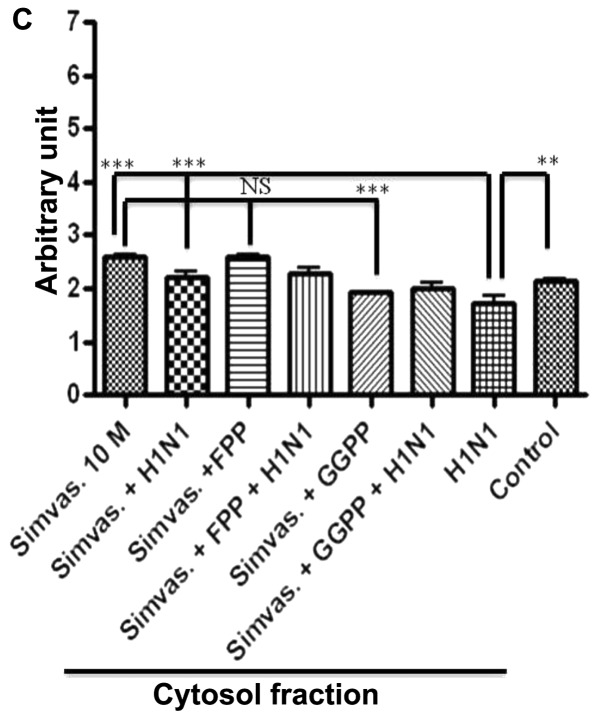
Modulation of the Rab7 protein translocation. (A) Confluent Madin Darby canine kidney (MDCK) cells were treated with simvastatin in the presence or absence of influenza A virus for 48 h. The effects of simvastatin were disrupted by 6 h pretreatment of exogenous geranylgeranyl pyrophosphate (GGPP), but not farnesyl pyrophosphate (FPP). The blots were typical of three independent experiments with similar results. (B and C) Density values of Rab7 protein isoprenylation are shown in membrane and cytosol extracts of treatments. Values show the density of the Rab7 protein bands normalized to the related housekeeping protein determined by Odyssey Infrared Imaging System and present the mean ± standard deviation (SD) of three different experiments from membrane and cytosol fractions of cell lysates. Data were analyzed statistically by Student’s t-test. (^***^P≤0.001; ^**^P≤0.01; NS, not significant).

Cell distributions of RhoA and Rab proteins in membrane and cytosol sub-cellular fractions with different treatments are shown in the western blots of Figs. 4A, 5A and 6A. Simvastatin treatment depleted these proteins from the membrane fractions, resulting in their enrichment in the cytosol section. The increase of RhoA and Rabs in membrane fraction for H1N1 samples and its depletion in cytosol fraction were evident. Treatment with Y-27632 as a selective inhibitor of Rho-associated protein kinases also resulted in a decrease in RhoA protein membrane translocation.

Statistical analysis verified the significant cell distributions (P≤0.001). Membrane localization of RhoA and Rabs was significantly decreased in simvastatin- and Y-27632-treated cells as compared to that of H1N1 (Figs. 4B, 5B and 6B) and their incidence in cytosol showed a significant increase compared to H1N1 (Figs. 4C, 5C and 6C). The effect of simvastatin was disrupted by exogenous FPP and GGPP, which increased the protein membrane translocation and was depleted in the cytosol section. GAPDH and Pan-cadherin as cytosolic and membranous housekeeping proteins remained unchanged after the treatments.

**Figure 7:**
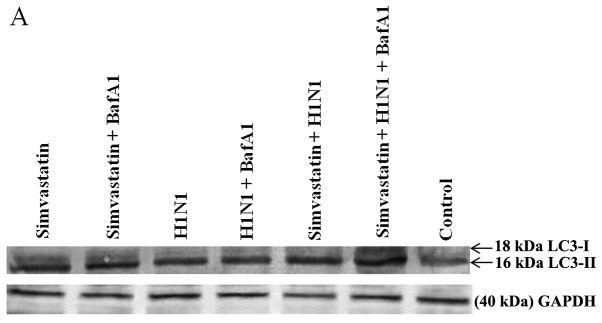

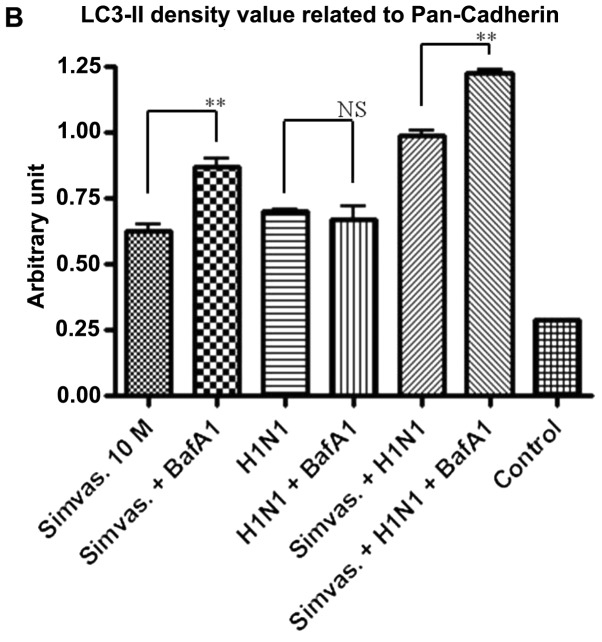
Modulation of the LC3 protein lipidation. (A) Madin Darby canine kidney (MDCK) cells were exposed to simvastatin and influenza A virus for 48 h with 6 h pretreatment of BafA1. Equal loading of protein samples was demonstrated with GAPDH antibody. (B) Density values of LC3-II protein was shown in the lysates of treatments. The values showed the density of the LC3-II protein bands normalized to that of housekeeping protein determined by Odyssey Infrared Imaging System and present the mean ± standard deviation (SD) of the cell lysates from three different experiments. The results are representative of the other statin treatments. Data were analyzed statistically by Student’s t-test. (^**^P≤0.01; NS, not significant).

Different distribution of lipidated LC3 (LC3-II) in different treatments are shown in the western blots in Fig. 7A. Based on statistical analysis (Fig. 7B), the membrane localization of LC3 protein in simvastatin and H1N1 samples was increased as compared to the control sample. Furthermore, LC3-II in the combined treatments of simvastatin with BafA1 was increased compared to the simvastatin treatment, but it was not affected in the combined treatment of H1N1 with BafA1. In addition, BafA1 increased the LC3-II to the highest value in the combined treatment of simvastatin and H1N1. GAPDH as a housekeeping protein was well detected and remained intact during the treatments.

### Lysosome localization detection

**Figure 8:**
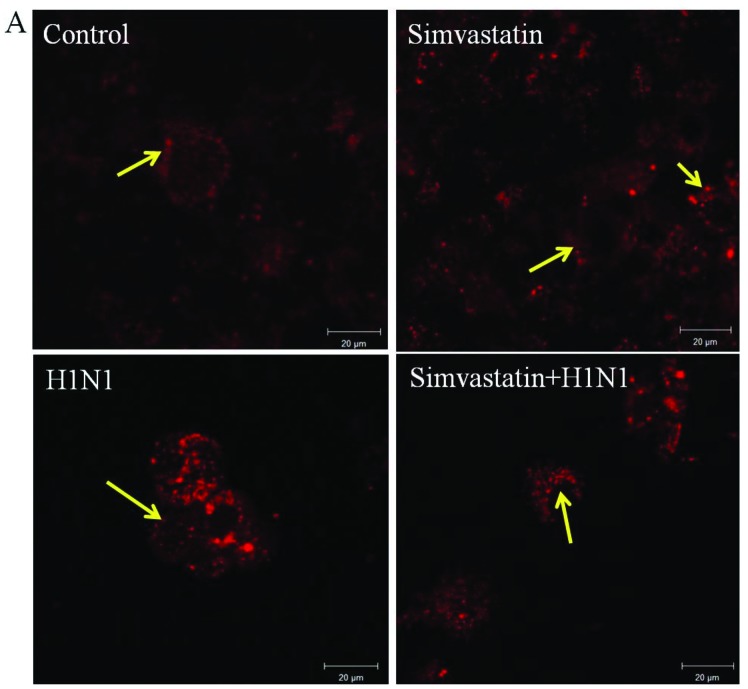

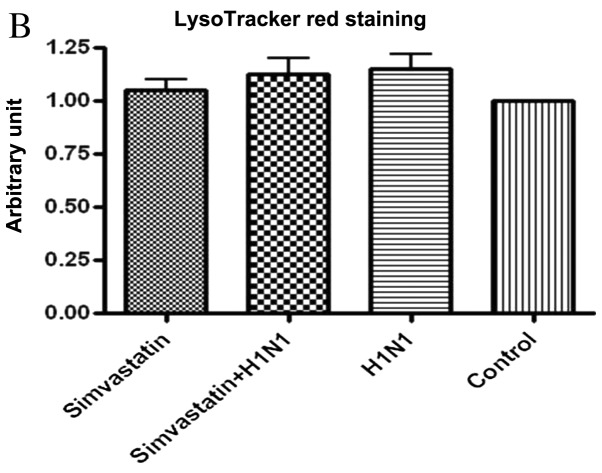
Alterations in lysosomal mass localization. (A) Madin Darby canine kidney (MDCK) cells were cultured on chamber slides in the presence of treatments as indicated in Materials and methods. The lysosomes were visualized by LysoTracker Red staining as a marker of lysosome activity. The arrows show the lysosomes localization around the nucleus. Images are representative of three independent experiments performed with similar results. Images were captured by 63× objective. (B) The graph shows the quantification of fluorescent intensities of lysosome activity in MDCK cells following treatments with simvastatin in the presence or absence of influenza A virus for 24 h. Data are the average of three independent experiments [mean ± standard deviation (SD)] normalized to the negative control sample, evaluated by LSM software and analyzed by the Student’s t-test.

MDCK cells in the control had a normal appearance in the punctate staining of lysosomes (Fig. 8A). Following treatment with simvastatin and H1N1 after 24 h, these localized compartments were elevated by an increase in spots and dense acidified lysosomes around the nucleus. The combined treatments of simvastatin and H1N1 showed a similar appearance in density values. These images were processed with LSM5 software and the average fluorescence intensities of three repetitions normalized to the control sample (mean ± SD) were used to quantify the results. The density of the acidified lysosomes increased similarly in all statins and virus treatments. Nevertheless, no significant differences were found in terms of lysosomal volume among mock-infected (control), simvastatin-treated, H1N1-infected and combined treatments (Fig. 8B).

## Discussion

Influenza A infection affects children, the elderly, as well as other high-risk groups (51,52). Available studies on the beneficial effects of statin treatments during the course of IAV infection are based on retrospective observational studies, which have shown that statins reduce morbidity during influenza pandemics as well as hospitalizations and deaths associated with seasonal influenza outbreaks (53,54). Statins potentially exert beneficial effects on influenza-infected patients by modifying the host response to the infection through mechanisms that are not connected with virus replication. Statins improve outcome in patients with sepsis and pneumonia (55,56). Furthermore, statin treatment of patients hospitalized with influenza is associated with a 41% reduction in a 30-day mortality, a reduction that may be attributable to previous vaccination or antiviral treatment (57). Authors of the abovementioned studies are in agreement that the benefits obtained from statins are due to their effects on the host response, and not the pathogen itself. This is of importance as, in any influenza patient treated with statin, the drug would affect the entire body, including cells and organs, in which no influenza virus may be found. However, the exact cell interactions and mechanisms of the effect of statins on IAV replication remain to be determined.

Previously, we showed significant reductions in the H1N1 viral titer in combined treatments with atorvastatin (45). The inhibitory effect of statins on the dysregulation of TNF-α and IL-6 cytokines caused by IAV was also demonstrated in CRFK cells (58).

The current study used different approaches to determine the effects of simvastatin treatment *in vitro* to reduce the ability of IAV to replicate. The concentration of 10 μM of simvastatin, which did not cause any toxicity on cell viability (Table I), was selected as the effective concentration (EC_50_) for antiviral experiments.

MTT and HA assays were conducted to verify the effect of simvastatin on the cell viability increment and viral load reduction. The metabolic effects of simvastatin were prevented by the addition of FPP and GPP (downstream products of the HMG-CoA reductase reaction) to the culture medium, but not by exogenous cholesterol, which is a major end-product of this metabolic pathway (Figs. 1 and 2). No significant effects of FPP and GGPP on HA and MTT results were found to be associated with the short-time incubations, which should be tested for longer incubation time treatments.

The antiviral effects of simvastatin on H1N1 viral load were examined using qPCR assay. In this test, the quantity of target genes was evaluated with reasonable accuracy by using a reliable standard. A standard curve, constructed from standard concentrations (data not shown) was used to determine the copy numbers of target genes related to the Ct value. The log_10_ copy numbers which were calculated from the concentrations against mean Ct values confirmed these significant decrements (Table II). Using ELISA confirmed that infection by H1N1 causes high expression levels of target pro-inflammatory cytokines in MDCK, while in all the combined treatments, the expression of these proteins decreased significantly and showed notable fold reductions compared to that of H1N1 (Table III). Therefore, it is possible to restrict immune system overexpression and lung inflammation by preventing the cell inflammatory responses to infection if proper treatment with simvastatin is applied.

The present study hypothesized that inhibition of RhoA and Rab protein prenylation by simvastatin could lead to inhibition of H1N1 transfer through the nucleus and cytoplasm by applying considerable changes in actin filament modulation and endocytosis.

RhoA regulates the contraction and relaxation of the actin cytoskeleton by coupling changes in the external environment leading to alterations in the actin cytoskeleton (59). Moreover, the actin cytoskeleton, which is imperative to viral translocation (60–62), is correlated with RhoA prenylation. Thus, the factors that restrict actin contractile function through inhibition of RhoA isoprenylation may have anti-influenza beneficial effects. The results of our study provided sufficient evidence to support the hypothesis that inhibition of RhoA and Rab protein membrane translocation as an underlying mechanism of action for simvastatin was responsible for the suppression of H1N1 replication.

Results of the present study revealed that H1N1 infection caused the activation of RhoA protein prenylation and induction of actin cytoskeleton remodeling. In addition, it was demonstrated that simvastatin reduced the replication of H1N1 by possible blocking of RhoA prenylation and membrane localization. It was shown that the cell distributions of the prenylated form of this protein in simvastatin-treated, Y-27632-treated and combined treatments of H1N1 were significantly different from H1N1 alone (P≤0.001) (Fig. 4). Furthermore, the present study demonstrated that simvastatin exerted direct cell effects on actin filament condensation (Fig. 3). During treatments using simvastatin, cell spanning actin fibers were reduced resulting in loosening of the actin cytoskeleton tightness and correlation. In H1N1-inoculated cells viruses induced actin filament condensation. This phenomenon suggests that H1N1 is involved in intermediate cell compartments requiring their movement through endocytosis, which is the verification of the critical role of simvastatin in regulating the state of intracellular actin filaments (63). Our findings are in agreement with the results from previous studies, which showed round and refractile morphology in lovastatin-treated cells (30 μM), which was due to the significant reduction in actin cables polymerization (17,64). These findings were also consistent with previous reports, which emphasized the role of Rho protein family in decreasing viral loads in an *in vivo* model of an acute HIV-1 infection, where it was suggested that changes in RhoA-controlled actin cytoskeleton rearrangements inhibit HIV-1 entry and exit from host cells (65).

It was also revealed that isoprenylation of RhoA facilitated the transport of H1N1 through an actin-dependent transport system. Inhibition of RhoA isoprenylation is necessary to suppress H1N1 replication. In addition, simvastatin can affect the upregulated RhoA pathway induced by H1N1.

Simvastatin treatments also led to a considerable decrease in Rabs prenylation, which affected H1N1 replication and promoted delocalization of Rab proteins from the endosomal membrane to cytosol. Therefore, the transport of viruses through early and late endosome vesicles may be affected. By contrast, H1N1 promoted their localization from cytosol to membrane. Density values of the expression of these proteins in different treatments showed significant differences in the cellular distribution of these proteins compared to H1N1 (P≤0.001). This effect was detectable in early and late endosomes at the level of Rab5 and Rab7 proteins, however, the expression level in early endosomes (Fig. 5) was more evident as compared to that of the late endosomes (Fig. 6). This could be deduced from the limited stability of the late endosomes (66). The simvastatin effect was significantly disrupted (P≤0.001) by adding GGPP but not FPP, whereby the GGPP reversed the statin effect and increased the Rab protein expression level in the membrane fraction. Inhibiting Rab protein prenylation appears to be another common mechanism of action for simvastatin as an anti-influenza agent. Besides H1N1, a previous study has shown the antiviral effects of simvastatin and lovastatin on HIV production in HIV-1-infected cells by affecting Rab11 geranylgeranylation (67). Furthermore, fluvastatin is also considered clinically useful for the treatment of diabetes by inhibiting Rab protein prenylation (68).

Other important pathways such as autophagy and lysis, which are involved in the pathogenesis of IAV (69), were also considered. This study highlights the effects of simvastatin on autophagy and lysis in the combined treatment with H1N1.

Modulation of autophagy by affecting LC3 as one of the most important molecules involved in autophagy may be an important mechanism to control cell component degradation (70,71). If cells cannot activate autophagy, protein synthesis predominates over protein degradation. By contrast, autophagy could be activated to guarantee survival of the cells (44,72). Previous studies have shown that during influenza infection, autophagosomes do not fuse with acidic compartments (70). Thus, accumulation of autophagosomes in H1N1-infected cells causes an increase in IAV survival (73). Therefore, inhibiting autophagy may inhibit virus replication (74). Viruses such as herpes simplex virus 1 (HSV-1), kaposi sarcoma-associated herpes virus (KSHV) and mouse herpes virus 68 (MHV-68) inhibit the autophagosome formation to escape autophagic degradation (69) while poliovirus, HIV-1 and mouse hepatitis virus (MHV) can block degradation of autophagosomes and use them as a platform to assemble their RNA complexes to increase the viral yield (70).

Therefore, blocking of autophagosome degradation would lead to gradual accumulation of autophagosomes and LC3-II (69). However, simvastatin increased autophagosome formation, leading to a certain degree of retardation in the maturation process of autophagosomes.

In this study, LC3-II membrane localization by western blot analysis showed that simvastatin was performed directly on autophagosome marker LC3 lipidation and its membrane localization and that the membrane localization of LC3-II was elevated. Thus it may be hypothesized that simvastatin treatments increased formation of autophagosomes. Moreover, the combined treatment of simvastatin with BafA1 as an inhibitor of lysosome activity increased the LC3-II level significantly (P≤0.01), which confirmed the results of simvastatin effects on LC3 lipidation. For H1N1 it was obvious that virus inoculation inhibited autophagosome degradation by LC3-II accumulation in cell lysate fractions and inhibited autophagosome maturation as the membrane localization of LC3-II was the same with or without BafA1 treatment. In the combined treatments of simvastatin and H1N1, the values showed increased intensities with the highest significant values amongst all the treatments (Fig. 7). The findings from this study were in agreement with those of a previous study on primary human lung mesenchymal cells, which emphasized that the relative amount of LC3-II is connected with the dynamic turnover of LC3-II via the lysosome activity (75). Thus, it is suggested that LC3 protein lipidation and membrane localization was upregulated by simvastatin and H1N1. However, simvastatin plays a direct role on LC3 lipidation through cholesterol depletion (42,76), although H1N1 involvement in autophagy seems to be far more complicated due to the effects of the upregulation of the expression of several autophagy-related genes that can increase the autophagic flux (74).

Evaluating lysosomal mass by fluorescent technique also showed that simvastatin and H1N1 exposure to the cells caused increments in the lysosomal mass, which, however, were not significantly different from those of the control (Fig. 8). Simvastatin induced lysosomal activity by increasing LC3 protein lipidation or promoting its localization, which is an important factor for autophagosome maturation. However, simvastatin treatment for H1N1 infection is potentially a double-edged sword as a decrease of cholesterol enhances autophagy, whereas if the reduction of cholesterol retards the maturation of autophagosomes, it may exacerbate the disease since the infectious agent is alive in the autophagosome.

In conclusion, findings from this study have demonstrated the ability of simvastatin to function in common cell pathways in order to block the virus-host interaction system, which renders it an effective agent against H1N1 infection. This process is the mechanism underlying the effects of potential anti-influenza agents such as HMG-CoA reductase inhibitors and other similar agents that may improve the development of new strategies to control H1N1 infection in influenza outbreaks. Since simvastatin exhibits antiviral effects by modulating common cellular machinery pathways, it is hypothesized that the mechanisms of the antiviral effects of simvastatin are not limited to its effect on RhoA and Rab proteins and thus downstream-related pathways in this context should be investigated. It is also worth considering the timing and/or duration of statin usage in relation to IAV infection, and to determine whether the treatment should be administered prior to infection as a preventative agent, or following onset of symptoms.

## Figures and Tables

**Table I:** MTT cell viability results following simvastatin treatments on MDCK cells.

Concentration (μM)	OD (mean ± SD) Simvastatin
20	0.27±0.19^a^
15	0.40±0.28^a^
10	0.78±0.19
5	0.79±0.12
2	0.83±0.10
1	0.93±0.07
Control	1.00±0.00

Values (averages of three independent experiments) showed cytotoxicity of different concentrations of simvastatin at different time points (24, 48 and 72 h) on MDCK cells (mean ± SD).

a Significantly different from values obtained for drug-treated compared to untreated samples (P<0.05).

OD, optical density; SD, standard deviation; MDCK, Madin Darby canine kidney.

**Table II:** Nucleoprotein and matrix 2 genes log_10_ copy numbers in different treatments.

	Log_10_ copy nos./μl (mean ± SD)		
	
Absolute quantification	Co-inoculation treatment	Pre-inoculation treatment	Post-inoculation treatment
NP gene			
Infected			
H1N1	11.644±0.008		
Simvastatin	11.283±0.002^a^	10.990±0.011^b^	11.451±0.027^b^
Amantadine	11.240±0.011^a^	11.303±0.053^a^	11.036±0.200^a^
M2 gene			
Infected			
H1N1	11.620±0.039		
Simvastatin	10.912±0.013^a^	10.548±0.001^a^	11.247±0.047^a^
Amantadine	10.821±0.050^a^	10.924±0.187^a^	10.902±0.136^a^

Real-time PCR quantification of viral NP and M2 genes showed a reduction in copy numbers of target genes in combined treatments of simvastatin and H1N1. Amantadine treatment was conducted as the control treatment. Data show mean ± SD of log_10_ copy numbers from two independent repetitions in duplicate.

a,b Significant decrements with P≤0.001 and P≤0.01, respectively, in the combination treatments compared to the H1N1 untreated sample.

SD, standard deviation. PCR, polymerase chain reaction. NP, nucleoprotein; M2, matrix 2.

**Table III:** TNF-α, IL-6 and IFN-γ protein reduction in MDCK culture supernatants following 72 h exposure time as compared to the H1N1 treatment.

Treatments	TNF-α fold reduction compared to virus	IL-6 fold reduction compared to virus	IFN-γ fold reduction compared to virus
Negative control	2.02	1.49	2.29
Simvastatin	3.05	1.49	1.67
Simvastatin + H1N1	2.44	1.61	1.57

Fold reduction of TNF-α, IL-6 and IFN-γ cytokine proteins as a ratio to H1N1 inoculation was determined by ELISA, (N=4–6) for 72 h incubation. MDCK, Madin Darby canine kidney; TNF-α, tumor necrosis factor-α; IL-6, interleukin-6; IFN-γ, interferon-γ.
